# CX3CR1 Is Expressed in Differentiated Human Ciliated Airway Cells and Co-Localizes with Respiratory Syncytial Virus on Cilia in a G Protein-Dependent Manner

**DOI:** 10.1371/journal.pone.0130517

**Published:** 2015-06-24

**Authors:** Kwang-Il Jeong, Peter A. Piepenhagen, Michael Kishko, Joshua M. DiNapoli, Rachel P. Groppo, Linong Zhang, Jeffrey Almond, Harry Kleanthous, Simon Delagrave, Mark Parrington

**Affiliations:** 1 Sanofi Pasteur, Research North America, 38 Sidney St., Cambridge, MA 02139, United States of America; 2 Genzyme, Department of Pathology, 5 The Mountain Rd., Framingham, MA 01701, United States of America; Imperial College London, UNITED KINGDOM

## Abstract

Respiratory syncytial virus (RSV) is the principal cause of bronchiolitis in infants and a significant healthcare problem. The RSV Glycoprotein (G) mediates attachment of the virus to the cell membrane, which facilitates interaction of the RSV Fusion (F) protein with nucleolin, thereby triggering fusion of the viral and cellular membranes. However, a host protein ligand for G has not yet been identified. Here we show that CX3CR1 is expressed in the motile cilia of differentiated human airway epithelial (HAE) cells, and that CX3CR1 co-localizes with RSV particles. Upon infection, the distribution of CX3CR1 in these cells is significantly altered. Complete or partial deletion of RSV G results in viruses binding at least 72-fold less efficiently to cells, and reduces virus replication. Moreover, an antibody targeting an epitope near the G protein’s CX3CR1-binding motif significantly inhibits binding of the virus to airway cells. Given previously published evidence of the interaction of G with CX3CR1 in human lymphocytes, these findings suggest a role for G in the interaction of RSV with ciliated lung cells. This interpretation is consistent with past studies showing a protective benefit in immunizing against G in animal models of RSV infection, and would support targeting the CX3CR1-G protein interaction for prophylaxis or therapy. CX3CR1 expression in lung epithelial cells may also have implications for other respiratory diseases such as asthma.

## Introduction

RSV is responsible for more than 500,000 emergency room visits and over 50,000 hospitalizations annually in the U.S. alone [1–3]. While a prophylactic antibody called palivizumab is available for infants at high risk of severe RSV, there is no vaccine and no specific treatment for this infection. Therefore, there is considerable interest in improving our understanding of the pathogen, its interaction with its target organs, and in particular the initial events of viral entry into cells.

The F protein of RSV interacts directly with nucleolin, a proposed RSV receptor, and mediates the fusion of the viral and cellular membranes, thereby initiating infection [4, 5]. The antibody palivizumab neutralizes RSV by binding to F, and the F protein itself is being investigated as a vaccine antigen [6]. Prior to membrane fusion, additional mechanisms appear to facilitate binding of RSV to cells. For example, heparan sulfate proteoglycans (HSPG) are used by RSV to attach to continuous cell lines [7]. An HSPG-binding region has been observed in the F protein and it has been proposed that this structural element is sufficient to attach virus to cell lines expressing HSPG [8]. The G protein of RSV, which was identified as the RSV attachment protein [9] has an analogous domain [10]. However, HAE cells are reported not to express HSPG [11] and while it has been hypothesized that G protein mediates attachment of RSV through its interaction with the host fractalkine receptor CX3CR1 [12–14], data to support this view were not based on observations in differentiated lung cells. Nevertheless, several observations bolster the case for a G-CX3CR1 binding interaction having an important role in RSV infections. For example, Choi et al. have shown that antibodies against the central conserved domain of G block its interaction with CX3CR1 expressed recombinantly [15], and Zhang et al. have further demonstrated that blocking the G-CX3CR1 interaction by vaccination against the same domain afforded protection against RSV in a mouse model [16] and that human sera from recently vaccinated or infected children inhibited the interaction in vitro [17].

CX3CR1 is known to be expressed in T cells and monocytes [18] as well as microglia [19, 20] and neurons [21], but its expression in differentiated HAE cells and its proposed role in RSV infection of these cells have not been described in the scientific literature. Here we use differentiated HAE cell cultures, immunofluorescence, confocal microscopy, and molecular virology to show that CX3CR1 is expressed in ciliated cells targeted by RSV and that an antibody to G protein, or deletion of G, inhibits virus binding to HAE cells and reduces viral replication significantly. We propose that these data, together with past findings on the interaction of G and CX3CR1 in other cell types, suggest a role for G in the interaction of RSV with HAE via CX3CR1. If this interaction is confirmed to be physiologically relevant, it may have implications for the development of future vaccines or therapeutics.

## Results and Discussion

We selected differentiated HAE cell cultures for our studies because they are made by seeding primary airway cells obtained from a human donor in a culture system which allows them to differentiate into cell types observed in the human airway epithelium [22] and presumably correspond more directly to human lung tissue than established cell lines. Mucin-producing goblet cells as well as motile ciliated cells (S1 Fig, S1 Movie) are observed in this model system, enabling studies of numerous respiratory pathogens, including RSV [11, 23–30].

**Fig 1:**
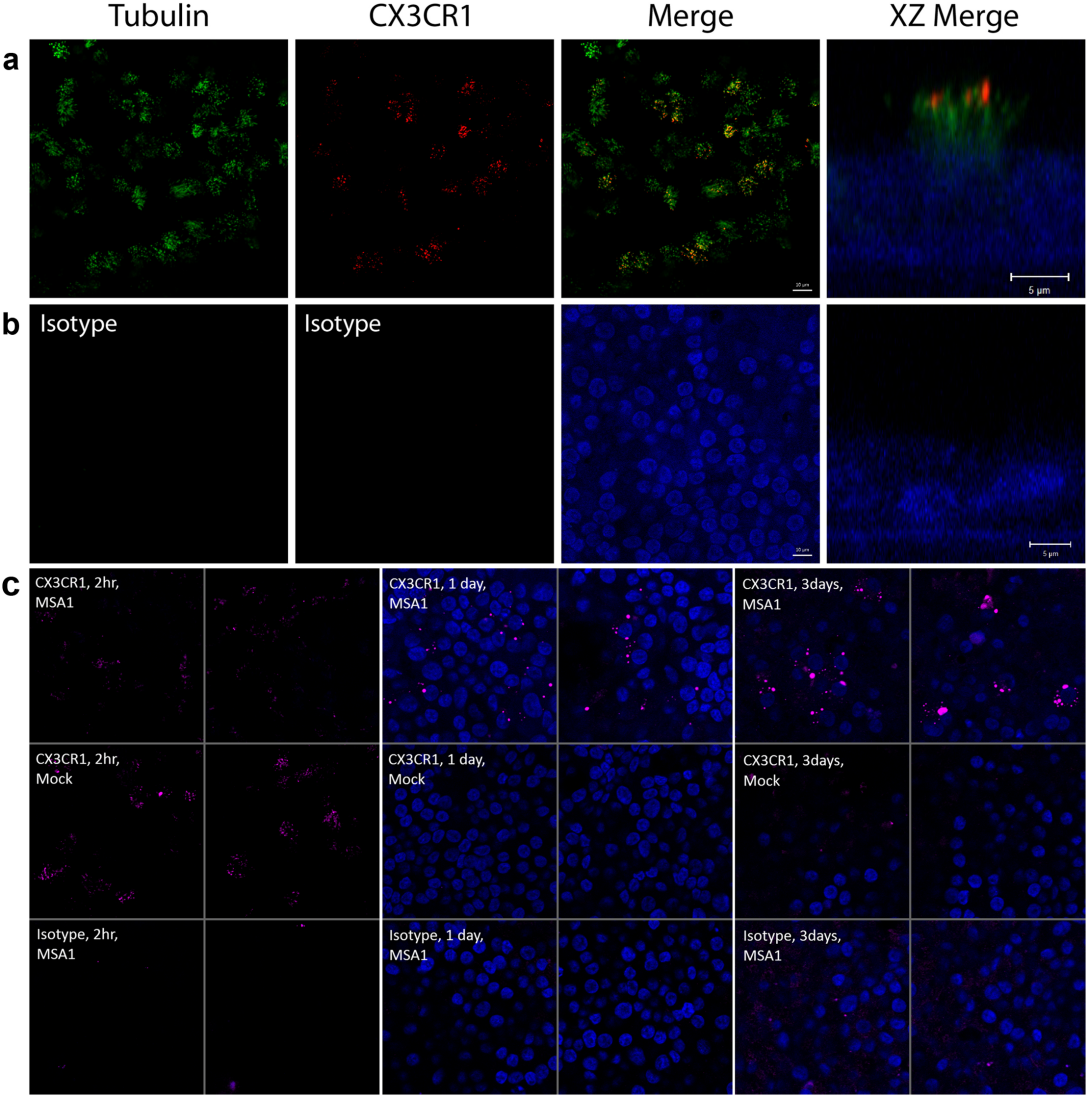
CX3CR1 is expressed in human airway epithelial cells and is localized to the motile cilia. HAE cells were grown in an air-liquid interface (ALI) culture system until differentiation was achieved, and imaged by confocal immunofluorescence. (**a**) Anti-β-tubulin (green) and anti-CX3CR1 (red) immunodetection of the cells seen *en face* are shown individually and merged (scale bar 10 μm). An xz plane image (XZ Merge) further illustrates the localization of CX3CR1 in motile cilia (scale bar 5 μm). Nuclei, stained with DAPI, are rendered in blue. (**b**) Immunostaining with isotype control antibodies confirms the specificity of the immunostaining. The merge image and XZ merge image are in planes that cross nuclei to confirm the presence of cells and the absence of non-specific immunostaining. (**c**) Confocal immunofluorescence images of differentiated HAE cells. Cells were either infected with RSV strain MSA1 (top and bottom rows), or mock infected (middle row). Images were acquired either two hours (left two columns), 1 day (middle two columns), or 3 days (right two columns) after infection or mock infection. CX3CR1 immunofluorescence is shown in (purple) (top two rows), or using an isotype negative control (bottom row). The cells were also stained for nuclei using DAPI (blue). Two representative images are shown for each condition tested. The 2-hour time point images are of an xy plane above the nuclei, intersecting the motile cilia. CX3CR1 immunofluorescence is clearly detected in the cilia of both infected and uninfected cells but is absent in the isotype control samples. The 1-day and 3-day time points are in an xy plane crossing the nuclei, 4.3 μm below the motile cilia.

Using confocal microscopy and immunofluorescence detection of β-tubulin and CX3CR1, it is apparent that CX3CR1 is expressed exclusively in ciliated cells, and is localized in motile cilia (Fig 1). Isotype control immunodetection shows minimal background fluorescence under the same conditions, and when using alternative secondary antibodies. At 1 day post-infection, punctate CX3CR1 immunofluorescence in the vicinity of the nuclei is seen in infected cells. This pattern is accentuated by day 3 as the structures positive for CX3CR1 appear to become larger (Fig 1C).

**Fig 2:**
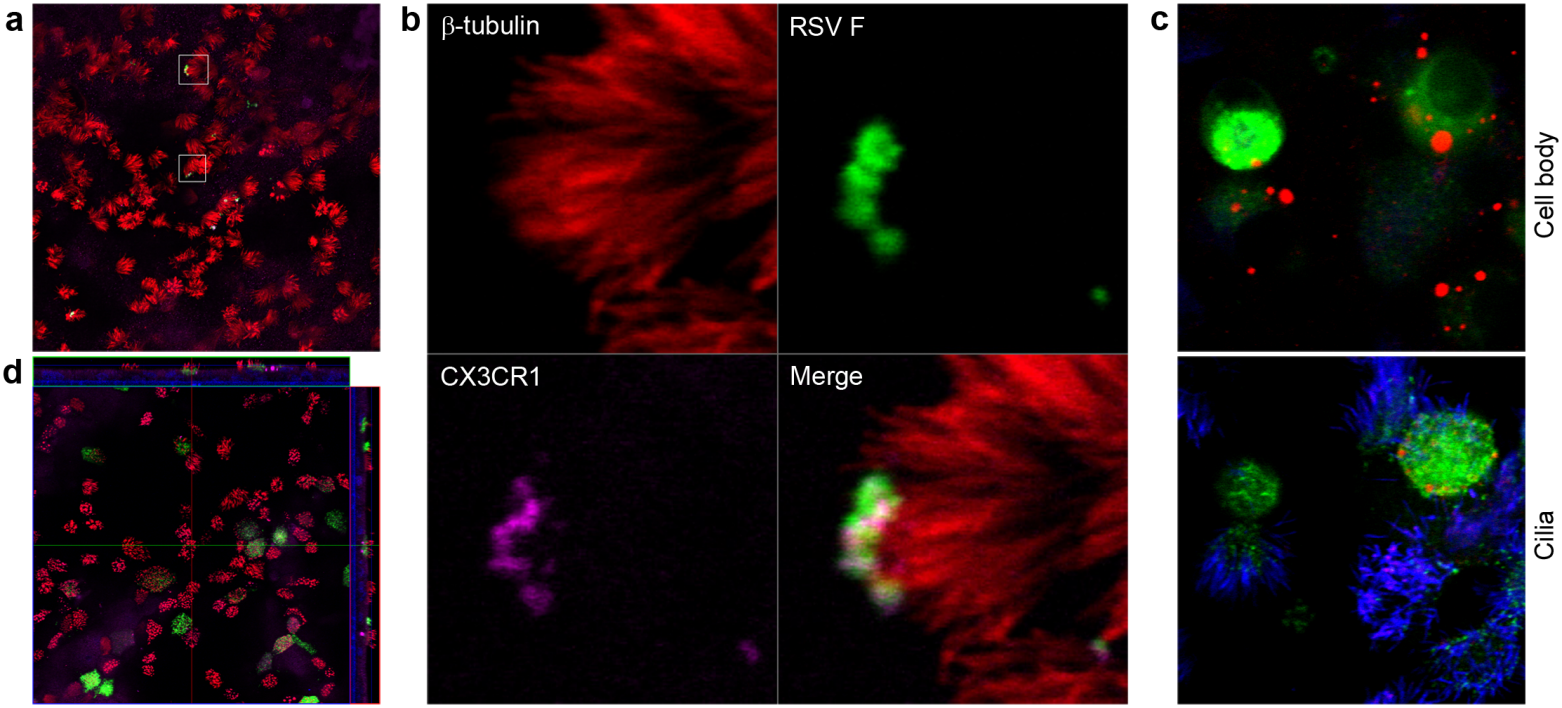
CX3CR1 in differentiated HAE cells interacts with RSV. Differentiated HAE cells, grown as in Fig 1, were incubated with RSV and imaged by immunofluorescence and confocal microscopy. (**a**) Binding experiment meant to visualize viral particles in association with HAE cells. Cultures were incubated 2h with RSV, then fixed and processed. RSV virions appear in green, β-tubulin is shown in red, and CX3CR1 is colored purple. Two regions of interest, representative of other RSV-bound cells in this image, are outlined with white squares. The top one is shown in expanded views in **b**, and the other one is shown in S2 Fig. Red, green, and purple images corresponding to β-tubulin, RSV F, and CX3CR1 respectively, are shown individually. Also shown is a merged image combining the fluorescence channels for RSV F, CX3CR1, and motile cilia. (**c**) Infected HAE cells were incubated for 3 days after infection and imaged using the same antibodies and fluorophores as in **a** and **b** but pseudo-colored differently: β-tubulin is in blue, RSV F in green, and CX3CR1 is shown in red. The two images are xy planes of the same sample separated along the z axis by 3.7 μm. The bottom image shows cilia and apical cell body, including some purple color indicative of colocalized tubulin and CX3CR1 immunofluorescence. The top image crosses the plane of the nuclei, below the cilia. Only infected cells (green) are surrounded by red-colored CX3CR1-positive circular features. Uninfected cells in the same sample and cells from uninfected control samples do not show these CX3CR1-containing structures (see Fig 1C). (**d**) Confocal immunofluorescence of HAE cells grown, differentiated, and infected for 3 days with RSV strain MSA1. Both *en face* (xy) and side (xz and yz) views are shown in this image. The xy plane of the *en face* view mostly cuts through the cilia of the cells, above the cell body. Nuclei are shown in blue using DAPI and, as in panel **a**, anti-RSV F protein is in green, anti-β-tubulin in red, and anti-CX3CR1 in purple. As in panel **c**, large ovoid structures positive for CX3CR1 immunofluorescence are seen in the side views and located near the nuclei of infected cells.

We then proceeded to investigate the interaction of RSV with HAE cells and CX3CR1. Confocal immunofluorescence imaging of RSV binding to cells reveals an almost exclusive interaction of virions with the cilia of ciliated cells (Fig 2A; S2 Movie). This pattern is consistent with the ciliated cell tropism of RSV observed when the infection is allowed to proceed for a few days (Fig 2D) [24]. Moreover, simultaneous detection of β-tubulin, CX3CR1, and RSV F demonstrates that virions and CX3CR1 are tightly colocalized at the distal end of cilia (Fig 2B; S2 Fig). As seen in Fig 1C, cultures allowed to incubate for 3 days after infection display a new distribution of CX3CR1 in RSV-infected cells in which the CX3CR1 immunostaining is now localized to one or more vesicles found near the nucleus (Fig 2C; S3 Movie). This redistribution of CX3CR1 appears as early as 1 day post-infection and is not observed in non-infected cells in the same culture or in control mock-infected cultures of the same age (Fig 1C).

**Fig 3:**
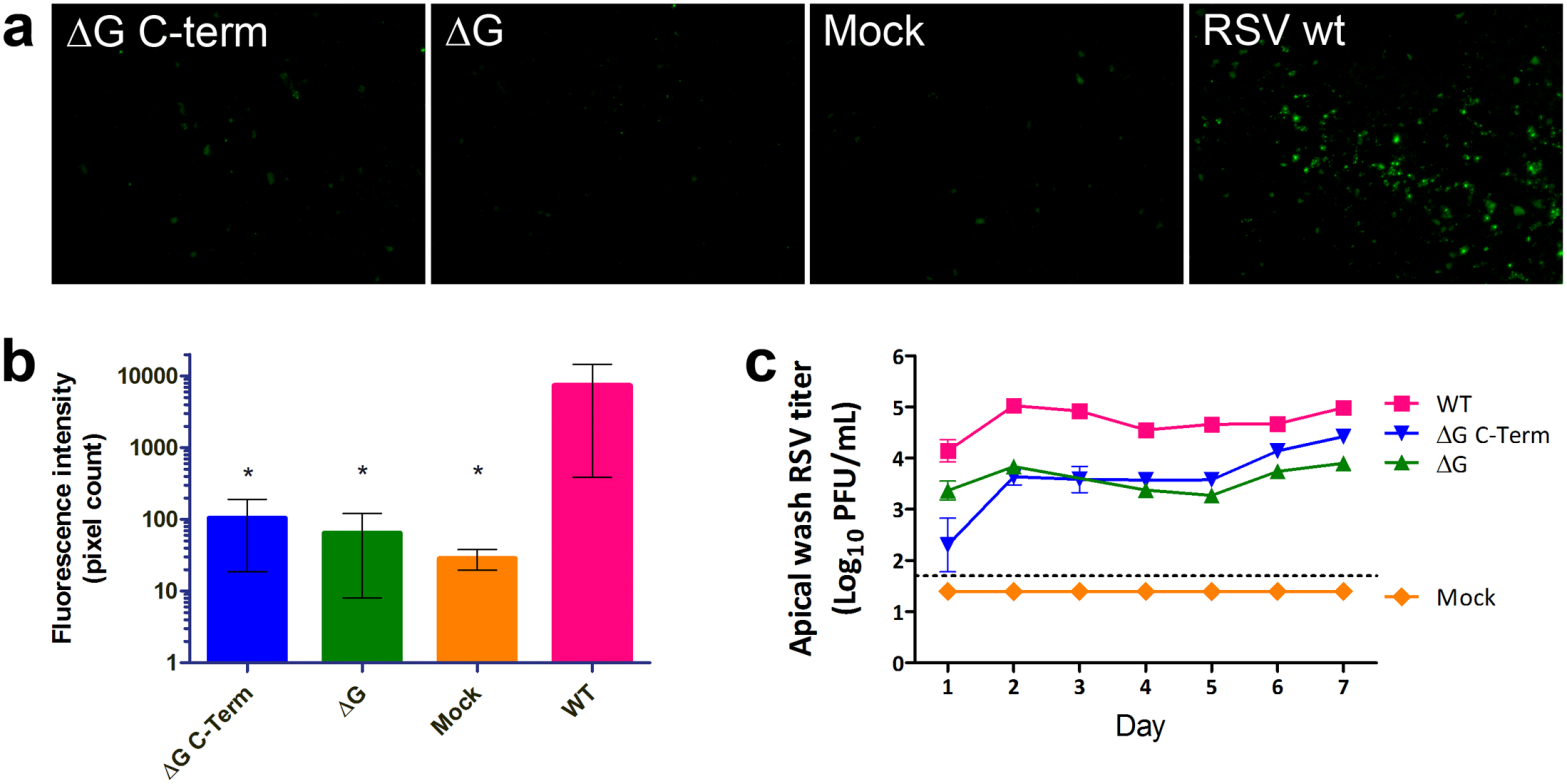
RSV interaction with HAE cells is strongly enhanced by G protein. Differentiated HAE cells were incubated with RSV wild type or mutants for 2 h at 37°C and either fixed and immunostained or incubated for several days to allow viral replication. (**a**) Anti-RSV immunofluorescence micrographs of HAE cells incubated 2 h with wild type (wt), or mutant RSV not expressing G (ΔG), or expressing truncated G that does not have the CX3C motif (ΔG C-term). (**b**) Quantification of virus binding using the images from 4 experiments, including those shown in **a**, by counting pixels of moderate to high brightness (gray values of 100 to 255). All groups exhibit significantly lower immunofluorescence intensity than wt. (**c**) Viral titers of apical washes taken daily from infected HAE cells. Titers of the ΔG mutant are significantly lower than wild type strain MSA1 on days 2, and 4–7, while the ΔG C-term mutant titers are significantly lower than wt on days 1–5. Error bars represent standard errors of the mean from triplicate measurements. * P < 0.05 by ANOVA.

To probe the RSV-CX3CR1 interaction further, we performed HAE cell binding experiments using RSV mutants. Starting from an antigenomic cDNA of the clinical RSV isolate MSA1, the entire G protein open reading frame or the C-terminal ~60% (from the CX3C motif to the C terminus) were deleted (S1 File). In a combined analysis of four experiments, the G protein deletion mutant exhibited a significant 116-fold reduction of binding signal, as measured by fluorescence intensity compared to wild type, and the G protein C-terminal deletion mutant showed a significant 72-fold reduction (Fig 3A and 3B). Counting of fluorescence spots also showed significantly reduced numbers in the G mutants (not shown).

We investigated the consequences on viral replication of the disruption of the G-CX3CR1 interaction. Mutant or wild type virus were allowed to replicate for 7 days during which daily washes of the apical surface of the cultures were collected and assayed for virus titer. This assay does not measure accumulation of virus in culture over time, as in a standard growth kinetics curve, but rather quantifies virus produced in consecutive 24-hour periods. As seen in Fig 3, viruses lacking full-length RSV G replicated at significantly (about ten-fold) lower rates throughout the experiment (Fig 3C). For all viruses the replication rate appears biphasic, with a first peak on day 2, a minimum on day 4 or 5, followed by a second increase on days 6 and 7.

**Fig 4:**
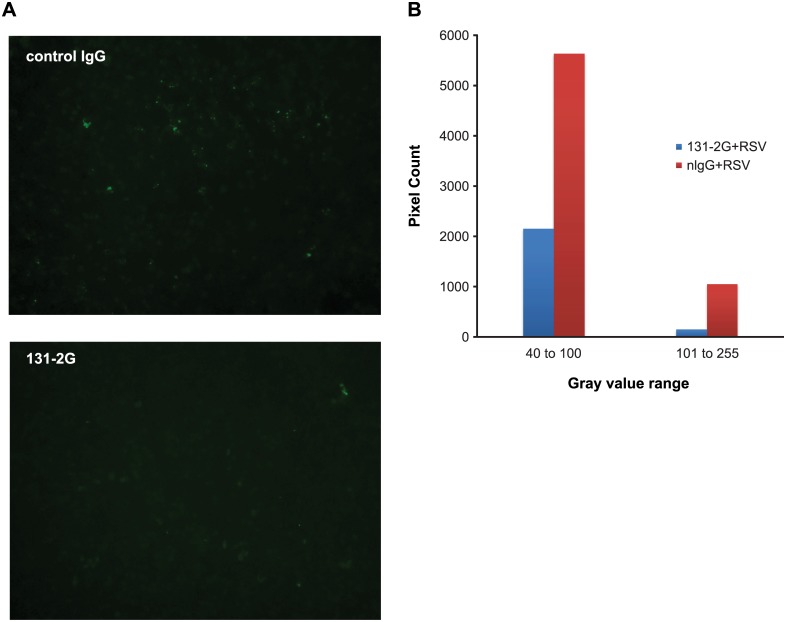
Antibody to G protein inhibits RSV binding to HAE cells. Wild type RSV was incubated with a negative control antibody or with 131-2G for 1h, and added to differentiated HAE cells for 2 h. (**a**) Fluorescence micrograph of cells fixed and immunostained against RSV F incubated with the indicated antibody. (**b**) Quantification of immunofluorescence intensity in a by counting pixels in moderate (40–100) or high (101–255) brightness ranges. Incubation with 131-2G significantly reduces immunofluorescence detection of RSV (*P* = 0.0001 by two-tailed t-test).

We also looked at the effect of the antibody 131-2G on virus binding to cells (Fig 4). This antibody has been shown to bind to a conserved epitope near the CX3C motif of the G protein and to inhibit the G-CX3CR1 interaction [12, 31]. Pre-incubation of RSV with 131-2G inhibits the interaction of RSV with HAE cells. These data indicate that the association of RSV with cilia is inhibited by disrupting the interaction of G protein and CX3CR1.

Using differentiated HAE cells, it has been reported that RSV exclusively infects ciliated cells and that infection leads to the production of a number of cytokines that have been observed in infected infants and in animal models [23–28]. Our data indicate that CX3CR1 localized in the motile cilia of differentiated HAE cells interacts with the G protein of RSV and facilitates binding of the virus to these cells, thereby enhancing infection efficiency. The previous observation of bitter taste receptors in the motile cilia of HAE cells supports the plausibility of finding other G-protein-coupled receptors such as CX3CR1 in these organelles [32].

A role for CX3CR1 in asthma has been proposed [33] and its expression in HAE may have implications for this and other respiratory conditions. A study of CX3CR1-expression in CD8+ T cells of infected infants has shown reduced CX3CR1 expression and an inverse correlation between expression level and wheezing duration [34]. In combination with a previous report that fractalkine is expressed in HAE cells [35], our observations indicate that both receptor and ligand are expressed in lung epithelium, suggesting that further investigations of the fractalkine signaling axis may yield additional information on lung physiology and disease.

Replication of RSV in HAE cells is significantly but not completely inhibited by disrupting the G-CX3CR1 interaction (Fig 3C). Even without its G protein, it is likely that RSV is still capable of infecting HSPG-expressing basal cells present in the pseudostratified epithelium of the culture system [11, 36], through interaction of F protein with HSPG and its receptor [4, 5]. The fact that the replication rate is biphasic with an increase after day 5 may be due to increased disruption of the apical cell layer during the course of the infection, allowing progressively more infection of basal cells [36] after an initial burst of replication in ciliated cells. In a healthy human host, an active immune system may take advantage of the reduced fitness of RSV lacking a functional G protein to better control its replication and prevent its transmission. This is consistent with the observation that G is necessary for efficient RSV replication in vivo, including humans, [37–39] and would explain why G is maintained in RSV despite its apparent redundancy in established cell lines.

There have been significant developments in recent years that may help in the development of a vaccine against RSV, as reviewed by Guvenel et al. and references therein [40]. The F protein has been a target of particular interest for vaccine design due to the discovery of new epitopes such as site Ø which are only present in the pre-fusion conformation of F and which elicit particularly potent neutralizing responses in vivo [6]. F is often considered a more promising vaccine antigen because its sequence is more conserved than that of the G protein, and because G is heavily glycosylated. However, G has been shown to confer protection in animal models as well or better than F. For example, virus-like particles (VLPs) comprising G protein made using a recombinant baculovirus expression system have been shown to be more protective in mice than similar particles comprising F [41]. Newcastle disease virus-based VLPs have also been used successfully to protect mice against an RSV challenge [42]. Nanoparticle vaccines comprising the central conserved domain of G have also been tested successfully in mice [43], potentially mitigating the problematic diversity of G protein sequences by focusing the immune response on a conserved motif directly involved in binding to CX3CR1.

Further studies will be required to confirm the role of CX3CR1 in the binding of RSV to cilia and in the infection process. However, published research on G as a vaccine antigen and past serological observations [44, 45] support the notion that targeting both F and G could afford better prophylaxis against RSV. While an antibody against the F protein is sufficient to achieve significant protection against hospitalization in infants, our observations suggest a new mechanism to explain why targeting both viral proteins could afford greater efficacy, regardless of whether the antibodies are provided directly or elicited by immunization.

## Materials and Methods

### Human airway epithelial cells

Primary human small airway epithelial cells (SAEC; CC-2547S, Lonza, Walkersville, MD) of a healthy 11-year-old donor were grown and differentiated in a humidified atmosphere (5% CO_2_, 37°C) as described previously [24, 26]. Briefly, reaching 75–80% confluence in T75 tissue culture flasks fed with small airway growth medium (SAGM; CC-3281, Lonza) supplemented with growth factors and hormones (ALI SingleQuots Kit; CC-4538, Lonza), the cells were dissociated with trypsin/EDTA and seeded on the semipermeable membrane of transwell culture inserts (6.5 mm diameter, 0.4 μm pore size; Corning-Costar, Lowell, MA) coated with rat tail collagen type 1 (BD Biosciences, Bedford, MA). When confluence was reached, an air-liquid interface (ALI) was created to trigger differentiation by removing the growth medium from the apical compartment of the culture inserts set in 24-well plates and replacing growth medium in the basal compartment with differentiation medium (CC-3281, Lonza) supplemented with the differentiation inducer (ALI SingleQuots Kit; CC-4538, Lonza). Thereafter, the differentiation medium was changed in the basal compartment every other day, and the apical compartment was gently washed with the culture medium once or twice a week to remove accumulated debris and mucus. The cells fully differentiate in 21 to 25 days of ALI culture into ciliated cells, goblet cells, basal cells, and non-ciliated columnar cells.

### Virus strains

Wild type RSV strain A1 (henceforth MSA1) is a clinical isolate. Its antigenomic cDNA was synthesized and ligated into a mammalian expression vector to generate the plasmid pMSA1. To create the G gene deleted (ΔG) and C-terminus truncated (ΔG C-term) constructs, fragments were synthesized spanning G and bordered by the naturally occurring, unique, restriction sites XhoI and BamHI in the MSA1 antigenome. The ~3 Kb ΔG fragment lacked the entire G gene including the gene start and end signal sites. The ~3.7 Kb ΔG C-term fragment lacked ~60% of the G gene C-terminus starting with the CX3C motif and including the stop codon and the gene end signal. The fragments were cloned into pMSA1 using the XhoI and BamHI restriction sites to generate the antigenomic cDNAs pMSA1ΔG and pMSA1ΔG C-term.

The MSA1, ΔG and ΔG C-term viral constructs were recovered from antigenomic cDNAs using a reverse genetics system similar to that described by Collins et. al.[46], twice plaque purified, and amplified by two passages on Vero cells. Viral preparations were confirmed by whole genome sequencing and titers determined by plaque assay on Vero cells.

RSV MSA1 and recombinant strains (ΔG, ΔG C-Term) were propagated in Vero cells (African green monkey kidney fibroblasts; American Type Culture Collection CCL 81) maintained in HyClone SFM4MegaVir (Fisher Scientific, Waltham, MA) supplemented with L-glutamine (GlutaMax; Life Technologies, Grand Island, NY).

### RSV binding and spread

For the virus binding assay, well-differentiated HAE cells were mock-infected or infected in triplicate with RSV strains (MSA1, ΔG, ΔG C-Term) prepared in differentiation culture medium at a multiplicity of infection of 3–5. After the incubation for 2 h at 37°C, the inoculum was removed and the apical surface was washed 4 times with culture medium. In addition, we performed a separate set of experiments in order to examine the role of the RSV G protein’s CX3C motif in viral binding and entry, by utilizing anti-RSV G protein antibody (131-2G; Millipore, Billerica, MA). The virus (MSA1 strain) was incubated with serial dilutions of 131-2G, ranging from 20 μg mL^-1^ to 400 μg mL^-1^, at 37°C for 1 h, and the virus-antibody mixtures were applied apically on the HAE cells for 2 h, and removed by washing with the culture medium. Virus particles binding to HAE cells were detected by immunofluorescence. ImageJ was used to quantify brightness in digital images as well as to count punctate fluorescent features.

For virus infection and characterization of replication, HAE cells were mock-infected or infected with RSV strains, and the apical washes were performed (3 times 120 μL, 15 min interval) daily on 1 to 7 days post-infection. Harvested washes were stored at -80°C until use for plaque assay (viral titration). In addition, sloughing and cilia beat frequency of HAE cells were examined in the microscope (Nikon Eclipse Ti-S) over time after infection. Infection patterns of RSV strains in HAE cells were monitored at various times after infection (2 h; day 1, 3, 5, and 7) by immunofluorescence.

### Immunofluorescence and confocal microscopy

HAE cells were fixed for 15 min with 4% paraformaldehyde (Electron Microscopy Services, Hatfield, PA) added in the apical compartment of the culture inserts, and permeabilized for 30 min with 0.25% Triton X-100 (Pierce, Rockford, IL) in phosphate-buffered saline (PBS). Following 3 washes with PBS containing 0.25% Triton X-100, the cells were blocked with Superblock solution (Thermo Scientific, Rockford, IL) for 1 h at 37°C. Next, primary antibodies prepared in Staining buffer (BD Biosciences, San Diego, CA) were applied on the cells overnight at 4°C or for 2 h at 37°C: anti-RSV F protein (clone 133-1H, mouse IgG2 conjugated with Alexa Fluor 488; Chemicon) for RSV-infected cells, anti-beta tubulin (clone TUB 2.1, mouse IgG1 conjugated with Cy3; Sigma-Aldrich) for ciliated cells, anti-Muc5Ac (clone 45M1, mouse IgG1 conjugated with biotin; abcam, Cambridge, MA) for goblet cells, and anti-CX3CR1 antibody (rabbit IgG; Sigma-Aldrich) for localization of CX3CR1, a proposed receptor for RSV (CX3C motif of RSV G protein).

After washing 3 times, appropriate secondary reagents (Alexa fluor-conjugated secondary antibodies or streptavidin, Molecular Probes) were applied for 1 h at 37°C: goat anti-rabbit IgG antibody conjugated with AF555 or AF647 for anti-CX3CR1 primary antibody, and streptavidin-AF350 for detection of anti-Muc5Ac primary antibody. Cells were washed 3 times, and counterstained with DAPI or TO-PRO-3 iodide (Molecular Probes). The membranes with HAE cells were cut from their supports and mounted on slides with antifade reagent (Prolong Gold; Molecular Probes, Eugene, OR). Fluorescent images were acquired using a widefield epifluorescence microscope (Nikon Eclipse Ti-S) with NIS-Elements (BR 3.10) software or with a laser scanning confocal microscope (LSM501META, Carl Zeiss) with Zen software (Carl Zeiss). The confocal microscope was equipped with 20x, 0.8NA dry or 40x, 1.2NA water immersion objective, and was configured for sequential channel imaging to minimize cross talk. Imaging parameters were established to ensure Nyquist sampling, and for a given staining combination, all imaging parameters were kept constant. Z-stacks were acquired from all samples to fully capture variations in staining along the apical-basolateral axis of the cells. Acquired images were subsequently examined and analyzed using Zen software (Carl Zeiss).

### Western blotting

Cell lysates from virus cultures grown for 6 days on Vero cells were subjected to SDS-PAGE, transferred to membranes and probed with a proprietary anti-RSV F antibody (#5353C7), or anti-M2-1 antibody (Abcam, ab94805), and incubated with a secondary goat anti-mouse alkaline phosphatase conjugate (Southern Biotech, 1030–01). Anti-G western blot was done with rabbit anti-RSV-G polyclonal (Sino Biological, 11070-RP02) and incubated with goat anti-rabbit secondary antibody (Abcam, ab97048). Bands were visualized using BCIP/NBT. The molecular weight marker used was Precision Plus Protein Dual Color Standards (Bio-Rad, 161–0374).

## Supporting Information

S1 Fig Immunofluorescence imaging of HAE cells confirms differentiation. HAE cells were grown in an ALI culture system and analyzed by immunofluorescence. Differentiation was confirmed by immunodetection of different cell types and susceptibility to infection by RSV strain MSA1. RSV F protein expression is detected by an anti-RSV F antibody (green). Anti-β-tubulin is used to identify the motile cilia of ciliated cells (red), and mucin-producing goblet cells are visualized using an anti-Muc5Ac antibody (blue). Extended spindle shaped Muc5Ac immunostaining is thought to be due to secreted mucus.
(TIF)

S2 Fig CX3CR1 in motile cilia of differentiated HAE cells interacts with RSV. As part of a binding experiment meant to visualize viral particles in association with differentiated HAE cells, HAE cells were grown in an ALI culture system, the cultures were then incubated with RSV for 2 hours at 37°C, fixed, and visualized by immunofluorescence and confocal microscopy. Two regions of interest are shown as white square outlines in Fig 2a. The top one is shown in expanded views in Fig 2b, and the bottom one is shown here. Red, green, and purple images of the same field of view, corresponding to β-tubulin, RSV F, and CX3CR1 immunostaining respectively, are shown individually. Also shown is a merged image revealing the colocalization of RSV F, CX3CR1, and motile cilia (tubulin). Virions appear to be associated exclusively with ciliated cells and to preferentially associate with CX3CR1.
(TIF)

S1 File RSV mutants show expected patterns of G, F, and M2-1 expression. Western blotting of Vero cell lysates infected with RSV ΔG, ΔG C-term, and wild type (MSA1). (**Figure A**) Detection of G using anti-G polyclonal antibody. (**Figure B**) Detection of F using anti-F antibody. (**Figure C**) Detection of M2-1 using anti-M2-1 antibody. Each Figure shows samples that were run on the same gel and irrelevant lanes are covered with white rectangles. Molecular weights in kD are shown in Figure A.
(PDF)

S1 Movie Differentiation of HAE cells can be monitored by observing the beating cilia of ciliated cells. In this video clip, HAE cells were allowed to differentiate in an ALI culture system for 21 days. As a result of differentiation, beating of the motile cilia of ciliated airway cells can be seen using bright-field microscopy.
(ZIP)

S2 Movie Three-dimensional video rendering of confocal image stack from a binding experiment measuring the association of RSV MSA1 to HAE cells. Cells were infected and processed as described in S2 Fig. Note the association of RSV staining with the apical region of ciliated cells. (Green, RSV anti-F staining; Red, anti-tubulin staining; Pink, TO-PRO-3.)
(AVI)

S3 Movie Video of a confocal three-dimensional model of HAE cells 3 days after infection with RSV strain MSA1, generated as described in Fig 2D. Note the presence of CX3CR1 staining in vesicles proximal to the nuclei of infected cells. (Green, RSV anti-F staining; Red, anti-tubulin staining; Pink, anti-CX3CR1; Blue, DAPI.)
(AVI)
