# Supplementary figures and images for: CX3CR1 Is Expressed in Differentiated Human Ciliated Airway Cells and Co-Localizes with Respiratory Syncytial Virus on Cilia in a G Protein-Dependent Manner
Source: PLoS One. 2015 Jun 24;10(6):e0130517. doi: 10.1371/journal.pone.0130517 (PMC4479564; doi:10.1371/journal.pone.0130517)

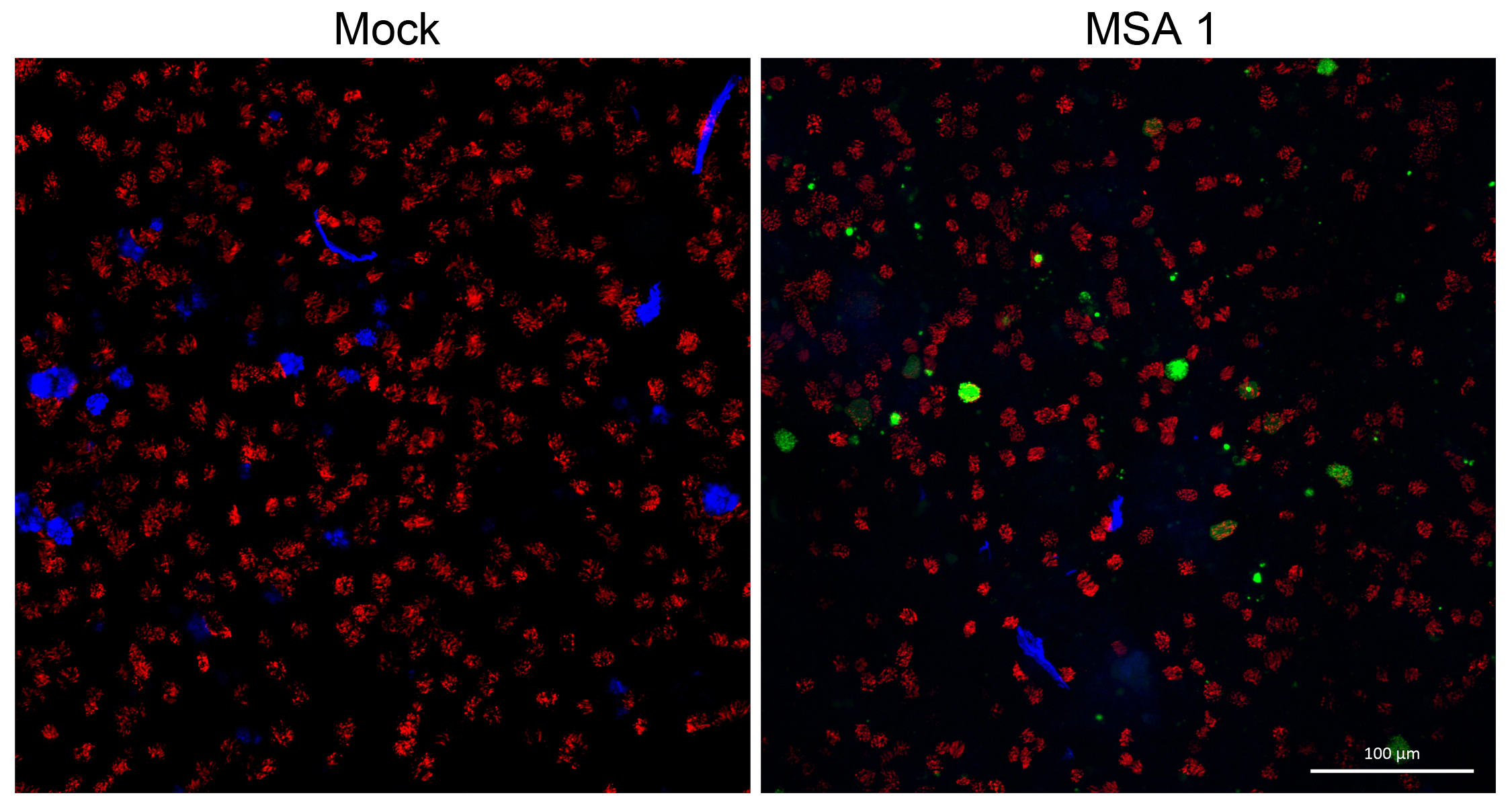

Supplement: S1 Fig — HAE cells were grown in an ALI culture system and analyzed by immunofluorescence. Differentiation was confirmed by immunodetection of different cell types and susceptibility to infection by RSV strain MSA1. RSV F protein expression is detected by an anti-RSV F antibody (green). Anti-β-tubulin is used to identify the motile cilia of ciliated cells (red), and mucin-producing goblet cells are visualized using an anti-Muc5Ac antibody (blue). Extended spindle shaped Muc5Ac immunostaining is thought to be due to secreted mucus. (TIF) [file pone.0130517.s001.tif]

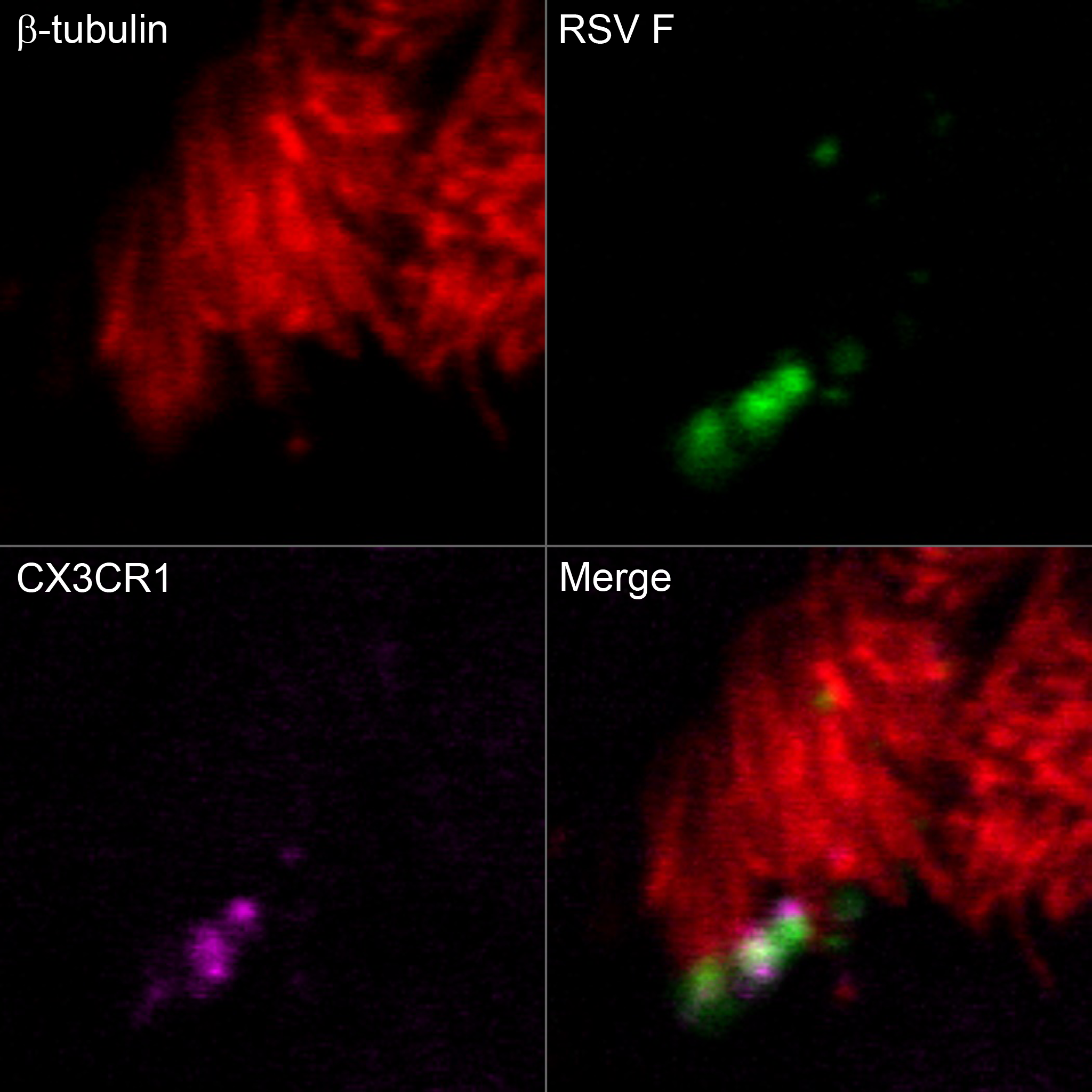

Supplement: S2 Fig — As part of a binding experiment meant to visualize viral particles in association with differentiated HAE cells, HAE cells were grown in an ALI culture system, the cultures were then incubated with RSV for 2 hours at 37°C, fixed, and visualized by immunofluorescence and confocal microscopy. Two regions of interest are shown as white square outlines in Fig 2a. The top one is shown in expanded views in Fig 2b, and the bottom one is shown here. Red, green, and purple images of the same field of view, corresponding to β-tubulin, RSV F, and CX3CR1 immunostaining respectively, are shown individually. Also shown is a merged image revealing the colocalization of RSV F, CX3CR1, and motile cilia (tubulin). Virions appear to be associated exclusively with ciliated cells and to preferentially associate with CX3CR1. (TIF) [file pone.0130517.s002.tif]

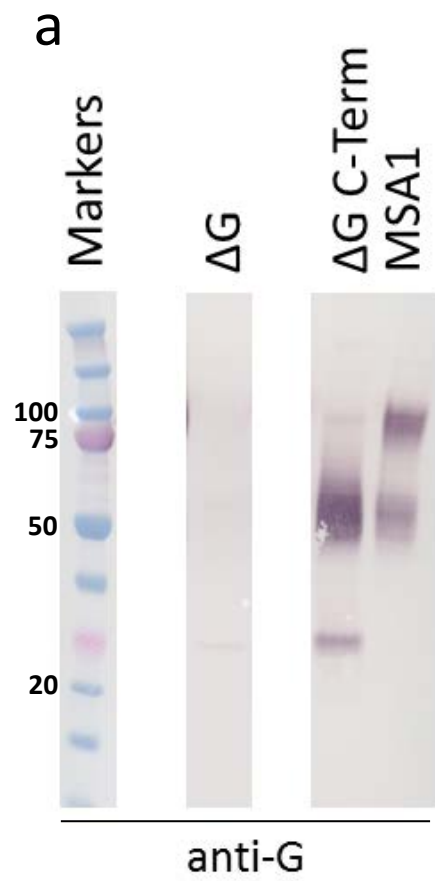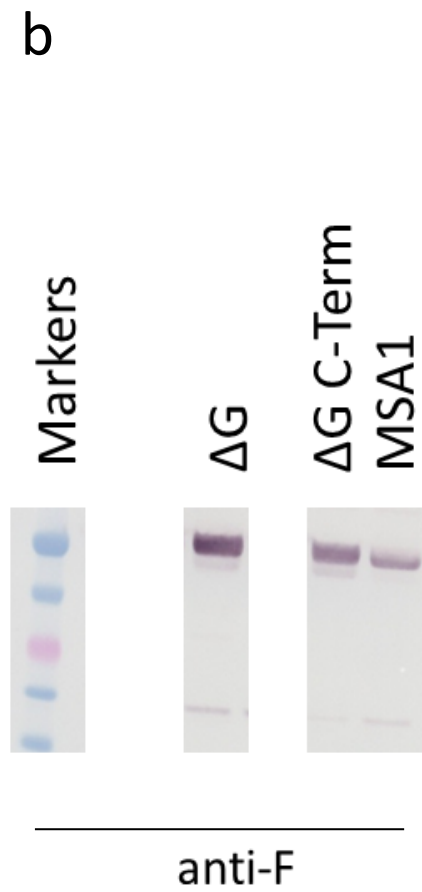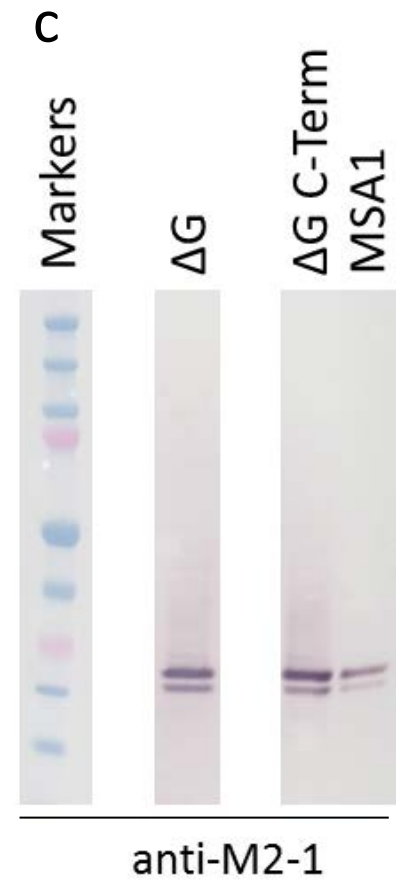

Supplement: S1 File — Western blotting of Vero cell lysates infected with RSV ΔG, ΔG C-term, and wild type (MSA1). (Figure A) Detection of G using anti-G polyclonal antibody. (Figure B) Detection of F using anti-F antibody. (Figure C) Detection of M2-1 using anti-M2-1 antibody. Each Figure shows samples that were run on the same gel and irrelevant lanes are covered with white rectangles. Molecular weights in kD are shown in Figure A. (PDF) [file pone.0130517.s003.pdf]
